# A brief note from Pakistan: reflections from a British psychiatrist

**DOI:** 10.1192/bji.2023.28

**Published:** 2024-02

**Authors:** Musa Basseer Sami

**Affiliations:** MRCP, MRCPsych, PhD, Clinical Associate Professor of Psychiatry, Institute of Mental Health, University of Nottingham, Nottingham, UK; and private practitioner, Nottingham, UK. Email: musa.sami@nottingham.ac.uk

**Keywords:** Pakistan, mental health services, polypharmacy, lower-middle-income countries, poverty

## Abstract

A British general adult psychiatrist born and trained in the UK, who also considers himself Pakistani, had the opportunity to spend 2 weeks running a psychiatric clinic in a remote hospital in the Punjab province of Pakistan. In this article he offers some reflections on the unexpected culture shock he felt, on the hospital system, the patients he treated and their resilience in such a poor country.

For the past 2 weeks I have been running a psychiatric clinic in a remote hospital in the Punjab province of Pakistan. Although I was born in the UK and have completed all my training there, I still consider myself very much Pakistani. I visited often from childhood and briefly studied here. Nonetheless, in practising psychiatry I have had what might be considered a culture shock. It may be a good time just as I am leaving to briefly pen my thoughts.

## Many patients, no records

The first thing one experiences (after the heat) is a high patient load. People are waiting at the door of the clinic, some have taken many hours to get there (as they tell me: ‘*Dhakey kha kha key*’– ‘having had to eat the pushes’). Some have come locally, some from the villages. I did not keep an exact count but I probably saw around 150 patients in 10 days and this was probably because the work was light in the month of Ramadan. There is no concept of preserved half hour or 1 hour appointments for new patients (which now seems to me such a wasteful luxury). Patients attend the clinic room one after the other, as one leaves another enters. These people are unwell, they are poor, I may not see them again and I know I need to get this right. For the first few days I begin to wish I had developed general practice skills and feel that I am distinctly unqualified for this work. Perhaps it is a mistake to come here. Maybe I won't help anybody.

The next thing I notice is the lack of documentation. There is none. If previously seen by a psychiatrist, patients come with their files, consisting of lists of prescriptions with a date. If lucky, the local doctor may have written down ‘paranoid thoughts’, ‘sleeplessness’, ‘tension’. I am working backwards from scrawled prescriptions which are all in brand names I don't recognise. There is a 1000 page directory next to me (which appears to be out of date) but I do not have the time to go through this, so Google and the staff around me become my steady friends. I cannot understand the prescribing regimes. It is not unusual to see patients concurrently on risperidone, quetiapine and ziprasidone. Bromazepam is often thrown in. I learn to work backwards quickly with the patient – were there psychotic features? Was the 25 mg of quetiapine given for agitation (*bey-cheyni*)? It begins to make sense and I become adept at doing it. I get frustrated with the lack of adherence and it takes a few days to understand that some people don't take the medication because of the cost but are too proud to say this. To be honest, I have never thought about the difference between Pramtec, Pramcit and Citalo (brands of citalopram). I realise it makes a difference. I also know, from local news stories and the papers, that not all of the medication contains adequate amounts of the active ingredient – so I let the patients guide me as to what works. I wonder why some of my local colleagues are prescribing vortioxetine and paliperidone.

Secretly part of me envies the lack of documentation. For me this is a very good system – how can I ever be held to account? If I do not document suicidal ideation then for the sake of posterity it doesn't exist. The conscientious part of me thinks I should document something, so I write a brief paragraph explain my findings and the future plan.

## The language of communication – and of illness

People speak in Urdu or Punjabi. Urdu is straightforward although I rarely take patient histories in the language so I find myself straining to translate everyday concepts. I never work out how to say ‘butterflies in the stomach’. Punjabi is, I realise, not a language but an expanse. With urban Punjabi I am quite good at working out what is going on. This gives me confidence. As a Punjabi who cannot really speak Punjabi, I have always been a bit ashamed of this. This new-found confidence gets shot through when I realise I cannot understand the Punjabi from some of the villages and I wonder if I am *desi* (authentic) enough.

I come across a completely new illness early on that I have never seen before. It starts with *kinchow* (a stretching) of the muscles of the shoulders, the back of the neck and the head. It feels like someone is pressing on the head. *Chakar aatey hein* (there is dizziness). There is abdominal pain and vomiting. Sometimes I will get told ‘*behosh ho jaatee hoon*’ (‘I fall unconscious’). They complain of *dorey* – an unspecified term that can mean anything from a full-blown epileptic seizure to a panic attack. Some of the physical symptoms are described so intensely I ask for a full panel of bloods and abdominal examinations. I find myself discombobulated – what is this strange multisystem organic illness that I do not even know how to begin to investigate? The penny drops when I ask about biological symptoms of depression – they are invariably present. But when I ask whether there are any worries the patients tell me there is nothing the matter. So then I ask them about their life – how is the family, who do they live with, any money problems? Tell me about early life. The clinical picture starts to come together. This is an internalised depression and anxiety with severe psychosomatic manifestations against a background of chronic stress. I suddenly feel sick realising how ubiquitous poverty is the backdrop to whatever I see. The hardship is so ever-present I wonder whether my patients even realise it. After having gone around in circles with three or four (mostly) women my head begins to hurt. I find Urdu versions of the PHQ-9 (for depression) and GAD-7 (for anxiety) and ask them to fill them in in the waiting room. These are completed with an endearing honesty. This is by far the most common presentation and is inevitably always severe. I find a YouTube video in Urdu on psychosomatic symptoms for psychoeducation and the consultations begin to become more straightforward.

## The cultural and political environment

Where I see trauma I see resilience. Where I see risk I see family. The easiest and most sustainable risk mitigation strategy (in a sea of risk – several young children, frank psychosis, overworked spouse) are these few words: ‘*aap key saath koi hona chaiya*’ – you should have someone with you. No problem. For some reason, in this very poor country, this is not such a knotty issue as it seems to be in the UK. I remember there are huge calls out from the big research funders on social connectedness and wonder whether they should come spend a couple of weeks in Pakistan.

There is political crisis in the country. The government changes while I am here. It is rancorous, emotions are high and it makes for damn good television. The usual actors are involved, with some broad swipes taken by different sides on the role of ‘the establishment’ and ‘foreign powers’. On the ground, everybody is aware of the situation. They keep up to date. I phone around some of my relatives in different cities and get the same pulse as I get here. No one trusts the system. They are all tired. No one cares.

## The patients I see

I see children. I do not want to see children – I am a general adult psychiatrist, my neurodevelopmental training post was in intellectual disability, but what am I supposed to do with these families who have brought their children to see me ‘*dhakey kha kha key*’? I think maybe I can take a history at least. The most common complaint is *ghosa* – anger. I stick to the basics and realise that many of these kids have autism. I try to avoid making the formal diagnosis, and contact local colleagues who specialise in child psychiatry in the country. It is satisfying.

I see frank catatonic presentations, severe psychotic states, plenty of neuropsychiatry. I worry about sending people back to the villages. They return a few days later having taken the treatment I advised. These patients respond well. Is this not what I came into psychiatry for? A select group of patients need ECT or clozapine. I end up phoning around the psychiatric community. The ECT machine in the next city is broken. I will have to send them to Pindi, which is 4 hours away. It is a logistical nightmare. I realise I am making a difference.

I wonder about our work in the NHS and the ‘culture of lament’ of under-resourced services. I wonder about the millions we spend on research that sometimes seems to help researchers’ careers more than psychiatric patients. I wonder about the brutal nature of global inequality and the fact that when all is said and done I am going to go back home. I will leave these beautiful eyes and smiles and the prayers on the lips of these truly thankful patients so very far behind.

## Data Availability

Data availability is not applicable to this article as no new data were created or analysed in this work.

